# Voice, access, and ownership: enabling environments for nutrition advocacy in India and Nigeria

**DOI:** 10.1007/s12571-024-01451-2

**Published:** 2024-05-03

**Authors:** Danielle Resnick, Kola Matthew Anigo, Olufolakemi Anjorin, Shilpa Deshpande

**Affiliations:** 1 International Food Policy Research Institute, Washington, DC USA; 2 University of Abuja Department of Biochemistry, Abuja, Nigeria; 3 Nutrition, Agriculture and Health Initiative (NAHI), Abuja, Nigeria; 4 Independent Research Consultant, Hyderabad, Telangana India

**Keywords:** Advocacy, Enabling environments, Governance, India, Nigeria, Nutrition policy

## Abstract

What constitutes an enabling environment for nutrition advocacy in low- and middle-income countries? While a sizeable body of scholarship considers the enabling environment for nutrition policy, we focus specifically on the necessary conditions for advocacy. We argue that three factors—voice, access, and ownership—provide a useful lens into the advocacy enabling environment. These are operationalized, respectively, as the space to articulate and frame policy positions, entry points to interact with policy decision makers, and the existence of committed decision makers rather than those responding to pressures from external actors. These three factors are explored vis-à-vis a comparative analysis of two federal democracies—India and Nigeria—that each have vibrant advocacy communities confronting persistent malnutrition. Drawing on more than 100 structured interviews with nutrition advocates, government actors, donors, and researchers in the two countries, we highlight the ways in which voice, access, and ownership interactively shape advocacy efforts. In doing so, we find that Nigeria has a less ideological approach to certain nutrition issues than in India but also perceived to be more beholden to external actors in defining its nutrition actions. Recent restrictions on freedom of speech and association shrunk the civic space in India but these were less problematic in Nigeria. In both countries, the multi-tiered, multi-party system offers many different points of access into the policy arena, with sometimes negative implications for coordination. Overall, the paper contributes more broadly to the literature on enabling environments by highlighting potential indicators to guide nutrition advocates in other settings.

## Introduction

Over the last decade, the nutrition advocacy community has expanded dramatically. Some of the most notable examples include the establishment of the Scaling Up Nutrition (SUN) Movement in 2010 and the International Coalition for Advocacy on Nutrition that emerged from the 2013 Nutrition for Growth Summit. To align with Sustainable Development Goal 2 on tackling “Zero Hunger”, an advocacy hub also has been established to bring together actors from non-governmental organizations, nutritionists, the private sector, and others to collaborate on improved nutrition and food security goals by 2030.^1^ As Pelletier et al. (2013) note, this emergence of nutrition advocacy has contributed to core elements of a nutrition agenda coalescing around key tenets, including the importance of the first 1,000 days of life, use of stunting as a central indicator, recognition of a life-cycle approach that is inclusive of maternal nutrition, and a growing focus on multi-sectoral strategies. 

However, not all advocacy efforts are equally efficacious due to both characteristics of the advocates as well as the enabling environment where advocates are operating.^2^ Gillespie et al. (2013: 553) define the enabling environment for nutrition as encompassing “political and policy processes that build and sustain momentum for the effective implementation of actions that reduce undernutrition.” Several useful case studies highlight that an enabling environment has facilitated the adoption and implementation of different policies (Haddad, 2013; Harris et al., 2016; Hodge et al., 2015; Kampman et al., 2017). Yet, enabling environments for nutrition policy adoption and implementation hold some key differences from those that facilitate nutrition advocacy. While advocates may be critical to nutrition policy achievements, their own actions may be constrained or propelled by a broader set of factors.

Specifically, this paper argues that three key factors shape the space for advocacy in general and for nutrition advocacy in particular: voice, access, and ownership. Voice refers to meaningful spaces for civic engagement, deliberation, debate and reflection about nutrition data, policies, and programming. Access encompasses the ability of advocates to reach those actors with the power and authority to make nutrition decisions, and it is often shaped by the landscape of political institutions. Ownership conveys the degree to which the public sector is genuinely committed to nutrition actions rather than responding to pressures from external networks and donors.

We demonstrate this through a comparison of nutrition advocacy efforts in India and Nigeria—two federal democracies where tackling malnutrition remains a longstanding concern despite several government initiatives. In Nigeria, the Federal Ministry of Health (FMOH) developed the 2014–2019 National Strategic Plan of Action on Nutrition aimed at reducing the number of under-five children who are stunted by 20 percent by 2018 (FMOH, 2014). The importance of tackling malnutrition is highlighted in the country’s 2016–2020 Agriculture Promotion Policy (FMARD, 2016) as well as the 2016 National Policy on Food and Nutrition (FMBNP, 2016). In fact, a recent review uncovered that there are at least 19 nutrition-relevant federal policies within the country that span multiple ministries (Vanderkooy et al., 2019). Nevertheless, according to the 2018 National Nutrition and Health Survey (NNHS), 32 percent of children under five are stunted and only 27 percent of infants between 0 to 5 months of age are breastfed exclusively (NBS, 2018). Nigeria also has among the world’s largest disparities at the subnational level in stunting and wasting among children under five years of age (Kinyoki et al., 2020). 

India’s nutrition record is equally complex. On the one hand, the country has the highest absolute number of malnourished children in the world (Sharma, 2019). The percent of children under five who are wasted is estimated at 17.3 percent and while the share of children under five who are stunted has decreased dramatically between 2005-06 and 2017—falling from 48 to 35 percent—it still exceeds the regional Asian average; in addition, more than half of women of reproductive age have anemia (Development Initiatives, 2022). The most recent, fourth round of the National Family Health Survey (NFHS 4) from 2019–2020, which was fielded before the onset of COVID-19 in the country, uncovered that several Indian states have experienced reversals in progress on child malnutrition (IIPS, 2019).

On the other hand, the country’s nutrition and food policy landscape is extremely robust (Vir et al., 2013). In 2013, under the Congress Party, the Government of India enacted the National Food Security Act (NFSA), which essentially aims to provide a right to food: “ensuring access to adequate quantity of quality food at affordable prices to people to live a life with dignity” (Government of India, 2013). The NFSA resulted in statutory support for several extant entitlement programs, including the mid-day meals scheme that provides free lunches to school children, the Integrated Child Development Services (ICDS), which offers health and nutrition services to young children and pregnant women, and the Targeted Public Distribution System (PDS) aimed at providing food to households below the poverty line (Pingali et al., 2017; Varadharajan et al., 2014). In 2017, under the government of the Bharatiya Janata Party (BJP), a multi-sectoral program on child undernutrition was approved and launched in 2018 as the National Nutrition Mission (POSHAN Abhiyaan), covering all 640 districts and focused on, among other goals, reducing underweight prevalence in children between 0–6 years of age from the NFHS levels by 2022 (Sharma, 2019). During the same year, the Government launched the Transformation of Aspirational Districts program, which targets 115 districts in 28 states and provides health and nutrition interventions to selected women and children, such as counselling on infant and young child feeding (IYCF), treatment of anemia, and iron supplementation during pregnancy (Kumar et al., 2020).

Advocates play an important role in these two policy contexts, but their strategies and impact are conditioned by the broader enabling environments in which they are operating. This paper explores which features of the enabling environment in these two countries facilitate or stymie nutrition advocacy efforts by drawing on more than 100 structured interviews in the two countries conducted over the 2019–2021 period. These interviews occurred with nutrition advocacy organizations working in the areas of IYCF and/or large-scale food fortification (LSFF), government stakeholders, donors, and researchers. Advocacy is defined as persuading decision makers to take targeted actions to support a specific policy (Williamson & Rodd, 2016). We define advocacy organizations as non-profit agencies whose core mandate involves promoting particular causes, ideas, and norms (Keck & Sikkink, 1998) or who participate in such promotional activities in addition to providing direct services, including technical training, community education, and program implementation (Kimberlin, 2010).

The findings show that factors that facilitate voice—captured by political pluralism and civic space—offer advocates opportunities to engage with a broad range of policy actors and the public. Access is measured by considering who are the “veto” players for certain nutrition policy arenas, inclusive of the lead ministries and those with budget authority. The existence of nutrition coordinating bodies and policy champions also shape access. Ownership refers to whether policymakers have sufficient financial means and extant policies and programs that can be leveraged by advocates to promote their objectives. Collectively, we find that during the period of study, the Nigerian context offers more voice than in India for advocates but also less ownership, with a widespread perception that donors are driving many of the nutrition policy thrusts. India, by contrast, has several large-scale entitlement programs that nutrition advocates can leverage for their proposed interventions. Access is a mixed picture in both countries; the federal context and the existence of several leading ministries and key policy champions provide multiple entry points into the policy sphere. The downside, however, is the potential for slower progress, duplication, or contradictory policy interventions. Extant coordinating bodies are viewed as having only middling efficacy, especially by those who are not members of those bodies.

While the paper draws on findings from Nigeria and India, the framework around voice, access, and ownership, has the potential to be used more broadly to assess the enabling environment in other countries for not only nutrition advocacy but also advocacy around environmental, agriculture, and even gender policy. The following section therefore reviews the domains of the framework before turning to the data collected in the two countries. Subsequently, we review the findings vis-à-vis each domain for the two countries. This is followed by a summary discussion that highlights that the different components of the framework must be weighted according to advocates’ ultimate priorities. Therefore, before concluding, we provide a set of guiding questions to facilitate future investigations in other country settings about the ways in which the extant enabling environment can facilitate, or stymie, nutrition advocacy. One caveat is that nutrition policy development and implementation are outcomes of an endogenous and dynamic relationship between advocates and the enabling environments in which they operate. For the purposes of gaining traction on which features of a country’s overarching enabling environment facilitate nutrition advocacy, the specific tactics of advocacy organizations to exert impact are not discussed in detail in this paper. However, a focus on advocacy actions for greater nutrition policy efficacy is examined in an accompanying paper (Resnick et al., 2022).

## Advocacy and enabling environments

Analyses of the enabling environment have become widespread and have contributed to a growing consensus on what is needed to gain momentum in the nutrition policy arena (Gillespie et al., 2013). However, much of this literature is focused on political commitment to nutrition *policy* rather than specifically to factors facilitating nutrition *advocacy* (Haddad, 2013; Harris et al., 2016; Hodge et al., 2015; Kampman et al., 2017). Indeed, the same political commitment may or may not have been influenced by the actions of advocates in different countries. Shiffman and Smith’s (2007) important framework highlights the presence or absence of civil society mobilization as one of 11 factors shaping the prioritization of maternal mortality on country policy agendas. However, they do not unpack the conditions that facilitate or suppress such mobilization.

Moreover, nutrition policy and the nutrition community are not homogeneous entities and span a variety of coalitions that focus on both nutrition-sensitive and nutrition-specific interventions. Policies also have varying levels of visibility, cost, and complexity and are often supported by different interest groups (Batley & Mcloughlin, 2015; Resnick & Swinnen, 2023; Shiffman et al., 2016). This, in turn, affects how easy or difficult it is to mobilize political support for policies and which institutions need to be engaged. Assessments that consider the enabling environment at large for nutrition policy fail to identify variations among different communities or policies.

We aim to build on this important literature while contributing a more refined focus by analyzing the enabling environment for nutrition advocacy—rather than policy—and considering different dimensions of the nutrition sphere. In focusing on advocacy, we identify three overarching conditions that are key for advancing progress: voice, access, and ownership.

### Voice

Voice is fundamental for advocates in any domain given their need to articulate a particular viewpoint and find allies to amplify their perspective. Several factors can contribute to the landscape for voice. One is the overarching political regime that prevails. The social movements literature points to the importance of open “political opportunity structures” that allow for engagement and dissent (Tarrow, 1994). Similarly, the Advocacy Coalition Framework (ACF), which focuses on the criteria internal and external to advocacy coalitions’ efficacy within particular policy subsystems, highlights that free and fair elections as well as freedom of association and expression are generally necessary to allow for advocacy coalitions (Sabatier & Jenkins-Smith, 1993). In the specific domain of nutrition, several scholars have made similar points about the need for open media environments, openness to the use of data and research, and few restrictions on associational activity (te Lintelo et al., 2016; te Lintelo & Pittore, 2020).^3^ Sufficient space for debating nutrition data and trends as well as the pros and cons of different policy options and identifying relevant narratives similarly relies on an open political system. More broadly, several studies suggest that democratic regimes are more strongly correlated with better food security and nutrition, and health outcomes because of their need to retain public support by catering to citizen’s welfare (Besley & Kudamatsu, 2006; Blaydes & Kayser, 2011; Fumagalli et al., 2013).

Democracies rely on multi-party competition and allow voters to assess candidates based on their proposed policies, thereby generating an in-built incentive for leaders to consider the different views of advocates on particular issues. Yet, this is most likely where parties have a programmatic platform rather than rely on personalistic appeals. In programmatic systems, there are typically distinct ideological platforms that undergird parties’ linkages with their constituents and have deep, historical roots (Kitschelt, 2000). In personalistic ones, individual characteristics of leaders—including charisma, background, experience, and connections—play a stronger role than ideology in distinguishing among parties (Mainwaring & Torcal, 2006). Programmatic systems can be beneficial to nutrition advocates when policy appeals closely align with particular parties’ agendas—especially the ruling party—but can be equally problematic when they do not.

Increasingly, it is recognized that while freedoms of association and expression cannot exist in authoritarian settings, democratic elections are not sufficient to guarantee those freedoms. In fact, the work on illiberal democracy highlights that democratic elections can coincide with closing civic space in order to justify the ideological stances of a ruling party (Zakaria, 1997). The legal system is the most effective way to reduce this civic space by imposing mandatory, burdensome registration requirements for civil society organizations (CSOs) on a regular basis that act as a form of surveillance, finance laws that limit CSO access to foreign funding, and selectively enforcing the law to fine, arrest, or threaten advocacy groups that advance messages anathema to the state (Christensen & Weinstein, 2013; Dupuy et al., 2016; Glasius et al., 2020).

### Access

An enabling environment for advocacy also requires access to important decision makers to engage in information sharing, consultation, deliberation, and partnership (Roggeband & Krizsán, 2021). One way to operationalize access involves identifying the number of political actors who have veto power in the specific nutrition domain under consideration and in the policy system more broadly. Veto players are the necessary and sufficient set of decisionmakers who need to ascent to a policy decision before it can move forward (Tsebelis, 2002). Such veto players can be determined by the broad range of political institutions that exist or by the specific mechanisms in the relevant policy domain. Sabatier and Weible (2007) draw on a similar concept in the ACF when they emphasize “policy venues” and note that advocates must consider both the number and accessibility of each type of venue. Political institutions can shape veto players at a systemic level, with parliamentary, democratic, and federal structures typically having more actors who have decision-making authority. Moreover, pluralistic policy arenas have more venues for engagement than those that are dependent on more corporatist arrangements whereby a narrow group of political actors, business groups, and unions jointly make decisions.

This therefore requires advocates to consider where, institutionally, policy decisions are made from both a *de jure* and de facto perspective. For instance, does the decision fall to a particular ministry or agency, or does it require legislative approval? Is it under a national or local mandate? If the latter, do local governments have sufficient budget and administrative autonomy to make and implement decisions? In Vietnam, Harris et al. (2016) find that while many nutrition functions were legislatively decentralized to provinces, the continued dominance of centralized planning and budgeting meant that the efforts advocates invested in building the capacities of local actors were less effective. By contrast, in Kenya, where a new constitution in 2013 led to political, fiscal, and administrative devolution, the implementation of the national Food and Nutrition Security Plan was delayed because all the newly created 47 counties needed to first align their county development strategies to it (Hodge et al., 2015). Generally, more veto players can result in a greater possibility of slower decision making and fragmented advocacy. At the same time, more veto players offer more points of entry into the policy arena, allowing advocates to try alternative strategies with different actors to identify the most effective influence strategy.

Given nutrition’s multi-sectoral nature, there is a large possibility for fragmentation without coordinating institutions. Such coordinating institutions can include horizontal bodies across sectors as well as vertical ones from the center to the local level. Critically, these need to be funded with clear mandates and adequate human resources (Haddad, 2013; Nisbett et al., 2014; Pelletier et al., 2012). In places as diverse as Peru, Senegal, and Uganda, placing a multi-sectoral coordinating body in a political office, such as the Prime Minister’s office, elevated its visibility (Kampman et al., 2017; Mejía Acosta & Haddad, 2014; Namugumya et al., 2020).

Brokers and policy champions are another well-recognized factor that facilitate access to nutrition decisionmakers (Cullerton et al., 2017; Mejía Acosta & Haddad, 2014; Nisbett et al., 2015). Brokers are organizations within advocacy networks that facilitate linkages among groups who might not otherwise be connected. Since advocacy success often relies on having a broad group of organizations working together, brokers play a critical role in amplifying advocates’ messages and expanding their reach (Cheng et al., 2021; Hervé, 2014). They also may have more power in shaping policy agendas and filtering information and resources to other members of the network (Cullerton et al., 2017). Policy champions are high-level individuals who may have both broad appeal to the public and can access important decisionmakers. In turn, this means that advocates should identify champions who can help gain policy traction in order to enhance their access to the policymaking sphere. However, there is less evidence about which types of champions are more advantageous to target. In some cases, high level decisionmakers, especially politicians, are needed to gain leverage for action (Fyall & McGuire, 2015; te Lintelo & Pittore, 2020). In other instances, mid-level bureaucrats can be more worthwhile to target because when and if politicians leave office, advocates must invest time in re-building legitimacy and alliances (Pelletier et al., 2013). In still others, engaging with celebrities or traditional authorities with high levels of public credibility may be more advantageous.

### Ownership

Ownership implies that advocacy efforts will be much more impactful in settings where there is already policy buy-in than in those that are going to require convincing and commitment. Several studies show that where nutrition agendas are imposed on governments, especially by external actors such as donors, they are less likely to be successful because governments’ ostensible rhetoric may not match with genuine interest or conflict with other priorities (Harris, 2019; Storeng et al., 2019). Two dimensions of ownership include government allocations of resources, as necessary, to the relevant policy area and the existence of policy documents or programs that advocates can tap into when advancing their positions. These are equivalent to Baker et al.’s (2018) categories of “operational” commitment and “institutional” commitment, respectively.

Operational commitment involves sustained allocation of financial, technical, and human resources. Where domestic resource mobilization is not possible in the short-term, the existence of willing donors to provide financing and technical assistance is critical for pushing forward the policy agenda. However, it creates power imbalances, may be limited to a donor’s funding cycle, and may encourage governments to focus resources on other priorities, thereby undermining the long-term buy-in and sustainability of interventions. To address this dilemma, Mkambula et al. (2020) argue that advocates who push for policies that require significant budgetary outlays can bolster their case and enhance sustainability by also providing recommendations about how governments can finance their policy interventions over the long-term. Similarly, the existence of a nutrition policy or nutrition-related strategy to guide actions and provide a rallying point for advocates is also key (Engesveen et al., 2009). Large-scale social welfare and entitlement programs into which governments have invested their political capital —such as Ethiopia’s Productive Safety Net Program, Brazil’s Bolsa Escola, or Egypt’s Takaful and Karama cash transfer programs—can also be a useful entry points for nutrition advocates.

## Data and methods

The framework described above is used to understand the enabling environment for nutrition advocacy in India and Nigeria. In doing so, we combine secondary data with results from structured interviews based on a common survey questionnaire conducted in each country. In Nigeria, face-to-face interviews occurred with 66 stakeholders between October and December 2019 while in India, interviews took place virtually with 36 respondents between July–September 2021 due to the Covid-19 pandemic. All interviews were coded and conducted using SurveyCTO, and the questionnaire included both close- and open-ended response options.^4^ In both contexts, the stakeholders selected spanned advocacy organizations, government officials, donor community, media, and the research community (see Table 1). Purposive sampling occurred with those knowledgeable about the two policy domains—IYCF and food fortification. We identified potential respondents from a longlist of stakeholders in each country based on a comprehensive stocktaking of the nutrition policy landscape and confirmation that the relevant organizations were still operational. The difference in respondents, particularly from government agencies in India, likely reflect that interviews in that country were conducted during the height of the Delta wave of the pandemic and therefore, officials were preoccupied with other priorities.^5^ Nevertheless, the range of respondents included encompass those highly involved in nutrition policy processes, increasing confidence that the views captured are reflective of expert viewpoints. Our ethical protocols entailed assurance of individual respondents’ anonymity, and therefore, quotes from interviews will be identified with a key informant interview number preceded by an “N” for Nigeria and an “I” for India. The affiliations of the sample are provided in Appendix 1.

**Table 1:** Distribution of interviewees

**Stakeholder group**	**Nigeria**	**India**
Advocacy organization	23	25
Government	28	3
Donor	4	3
Media	3	2
Research community	8	3
Total	66	36

**Geographical distribution**	**Nigeria**	**India**
*Federal*	26	25
*State*	40	11
Total sample size	66	36

In each country, interviews focused on engaging with both actors at the national level and at the subnational level. In this regard, respondents were selected not only in the capital cities of Abuja and New Delhi but also in Kano and Kaduna in Nigeria as well as Bihar and Uttar Pradesh in India. This enabled us to ensure that the findings were broadly representative and not just reflective of national perspectives. The choice of states reflected high levels of both nutrition needs and advocacy activities. For example, in Nigeria, Kaduna and Kano fare even worse than the national average on many key metrics of child malnutrition. While malnutrition indicators in other states of northern Nigeria are even worse (Amare et al., 2018), many of those states are located in conflict-affected areas that make it difficult to safely implement advocacy activities. By contrast, Kaduna and Kano attract many advocates in the nutrition arena, and overall donor investments to address malnutrition in Nigeria are among the highest in these two states (World Bank, 2018). In India, stunting rates in children under five years of age in Uttar Pradesh—India’s most populous state—exceed the national average (Mani et al., 2017), and the state has among the country’s highest rates of maternal mortality (Kumari et al., 2019). Bihar has similar levels of stunting with two-thirds of women between 15 and 49 years and 87 percent of children under the age of three years suffering from anemia (Kathuria & Khanna, 2014). Despite some improvements in economic growth and governance since the mid-2000s, it continues to have India’s lowest ranking based on the country’s sub-national human development indices (Global Data Lab, 2021).

As seen in Table 2, the advocacy organizations in our sample predominantly prioritize infant, child, and maternal nutrition and/or addressing micronutrient deficiencies, and a majority of advocacy respondents have worked in their respective organizations for more than three years. This increases our confidence that they are not only knowledgeable about their organizations’ advocacy efforts but also about IYCF, LSFF, or both.^6^ Since these two domains are quite distinct in terms of policy development and fall under the mandate of different government agencies in both countries, we obtain a more nuanced understanding of debates and priorities within sub-communities of nutrition advocates, highlighting which aspects of the enabling environment are cross-cutting for these advocates and which are specific to the policy domains that they address. The two domains are also qualitatively different in terms of the institutions that need to be mobilized, the politics of winners and losers, and the resources required; as such, examining them in tandem provides a more holistic understanding of advocates’ role in the nutrition policy process and what enabling factors enhance or undermine their efficacy.

**Table 2:** Distribution of advocacy organizations by tenure length and priority area (%)

**Length of time working at organization **	**Nigeria**	**India**
Less than 1 year	4.2	0.0
1 to 3 years	33.3	0.0
3 to 7 years	41.7	40.0
More than 7 years	20.8	60.0

**Which health and nutrition issues does your organization prioritize for advocacy?**^a^	**Nigeria**	**India**
Infant & child nutrition	75.0	92.0
Maternal nutrition	54.2	84.0
Micronutrient deficiencies	62.5	80.0
HIV/AIDS	16.7	16.0
Malaria	12.5	20.0
Food access	29.2	76.0
Non-communicable diseases	8.3	32.0
Food safety	25.0	60.0
Water and sanitation	33.3	52.0

Number of advocacy organizations is 24 in Nigeria and 25 in India

^a^Shares total more than 100% because respondents could choose more than one priority

## Findings

### Voice

Both Nigeria and India are multi-party electoral democracies, but certain restrictions on civil liberties, including freedom of expression and association, have become more pronounced in recent years. This change has been more pronounced in India—which has seen its rankings on civil liberties decline notably since 2019—than in Nigeria, where civic space comparatively always has been slightly more restricted (Repucci & Slipowitz, 2021). Several advocacy organizations highlighted that India’s democratic environment was important to their activities (I15, I16). As one respondent said, “The government has a democratic mandate to take care of health and nutrition” (I15). Moreover, the diversity of the political environment ensures that an advocacy organization can always find a receptive actor, either in the opposition or in government (I2): “Diversity of political environment means that some political party either in opposition or government is receptive, this facilitates advocacy.”

In Nigeria, however, many nutrition advocates noted that elections created some challenges for their efforts. One challenge is keeping IYCF, which is a relatively low visibility public good, on the political agenda when voters really want to see more tangible investments: “There is a myriad of things and from the political angle, investing in Nutrition (IYCF) does not bring 'quick returns' as opposed to maybe CMAM [Community Management of Acute Malnutrition] or road construction. Even for health, on a scale of prioritizing things, nutrition will never come above immunization” (N11). A second challenge is maintaining momentum as new political leaders or ministers arrive in office: “I think first of all, nutrition policy is often influenced by the agenda of the government administration that is in power, but there are many things that motivate their agenda and nutrition is not always the priority. So, the fact that administrations change every four years, so if you have been doing advocacy and gaining traction of some sort, there is a chance that at the point when you are about to reap the reward of the advocacy you have done, that the administration will change and you will have to start all over again” (N63).

Nigeria’s two main political parties—the People’s Democratic Party (PDP) and the All Progressives Congress (APC)—are mostly distinguished by the personalities of their leaders rather than ideology, with politicians engaging in frequent party switching when it benefits their individual careers (Fashagba, 2014). By contrast, India has a mass party system with more programmatic interests. The current ruling party, the BJP, emerged from a Hindu nationalist movement and, according to scholars, has promoted policies that largely support a *Hindutva* populist strategy (Ganguly, 2021; Jaffrelot & Tillin, 2017). In this respect, the current ruling party’s Hindu nationalist stance circumscribes certain recommendations by nutrition advocates who promote animal-sourced nutrients. One example relates to political resistance to some advocates’ recommendations to provide free eggs to children, including through government-run day care centers known as *anganwadis*, in areas where there is high protein deficiency. Eggs are viewed by some upper-caste adherents to Hinduism as a type of meat product that should be eschewed to maintain purity (Chatterjee, 2015; Srinivasan, 2023). As one respondent noted, “With the changing political economy of the country, certain suggestions don’t go down well such as specific food supplements—particularly non-veg such as eggs and beef” (I4). Another noted the problem of ideological politics: “For example, the discussion around provision of eggs in *anganwadis* seems to be determined by religious ideology” (I20) while still another stated, “Currently, government is not open to civil society or external voices unless they align with their ideological philosophy on nutrition” (I21).

For similar reasons, the legal framework in India at the time of field work was also circumscribing advocacy efforts. Collectively, interviewees pointed to three sets of legislation that affected their ability to operate. The first and most mentioned is the Foreign Contribution Regulation Act (FCRA). Originally promulgated in 2010, the FCRA regulates access to foreign funds for associations, as well as companies and individuals, and prohibits receipt of such funds if they may undermine national interests. In September 2020, the FCRA was further amended with even stricter provisions. This included governmental oversight, extra regulations and certification processes, and only allowing non-governmental organizations to receive foreign contributions to support 20 percent of their administrative expenditures, rather than the 50 percent allowed under the previous version of the Act. The Act is considered to have an overly broad definition of the “national interest”, which can be tantamount to any activity an organization conducts that could be conceived by the sitting government as political or threatening.

Many organizations focused on the limitations created by the FCRA restrictions. International organizations felt a sense of suspicion by the government and limitations on their ability to advocate in much depth. Domestic organizations noted how receiving funding, especially for administrative expenses, is quite limited by the FCRA. One respondent noted that “Being an international agency is a limitation” (I6) while another observed that “[The] government is closing the door to work with international NGOs” (I7). Since the FCRA also restricts sub-contracting, it creates particularly difficult circumstances for smaller NGOs that work collaboratively with larger ones (ICJ, 2020).

The second legal act relevant to nutrition advocates is the Unlawful Activities (Prevention) Amendment Act (UAPA). Originally developed in 1967 as an anti-terrorism law to prevent unlawful activities, the Act’s Amendment in 2019 allows for a more expansive definition of terrorism that can be used to curtail the right to free speech and the right to dissent. A third is the Citizens Amendment Act (CAA), introduced in 2019 and representing the culmination of a campaign promise made by the ruling BJP during parliamentary elections in both 2014 and 2019. This law introduces a religious basis for Indian citizenship, enabling illegal, non-Muslim immigrants from neighboring countries to gain Indian citizenship faster than illegal Muslim immigrants (Akins, 2020).

As a result of UAPA and CAA, narratives around rights-based approaches to nutrition are more contentious. After citizen protests in Uttar Pradesh against the CAA that generated a heavy-handed response by the government, including arrests of some protestors under the UAPA, the ability to exercise free speech became much more constrained. As one respondent for an advocacy organization there noted, “We are not free to talk about rights, to raise demands, to advocate to organize public rallies like we did before. Before the CAA, our strategy was completely different but now it is not possible” (I31). The CAA can be particularly problematic for organizations with an expansive target population, including children of migrant workers and those from religious minority communities (I9).

Nutrition advocates in Nigeria did not mention similar concerns about the legal environment and, in fact, as discussed in more detail later, have an even greater dependence on external funding and overseas partnerships. Worryingly, however, a restrictive NGO bill that was originally introduced in the House of Representatives in 2017 to regulate NGO and CSO activities—and dropped after widespread protest—was reintroduced in mid-2022 after fieldwork for this paper was completed.^7^ The passage of such a bill could potentially have the same effect on dampening nutrition advocacy in Nigeria as it has in India.

### Access

Since Nigeria is a federal system, nutrition policies need to be ratified at the state level and therefore, national policies may not be uniformly implemented at the subnational level (Vanderkooy et al., 2019). The country has 36 states, plus the federal capital territory of Abuja, as well as 774 local government areas (LGAs). Nigeria’s federal system contains a two-tiered legislature comprised of a Senate and a House of Representatives. Within the federal House of Representatives, standing committees are used to consider policies relevant to their sectoral expertise. The standing committee will both identify the sectoral projects to be implemented in the coming year and should monitor the ministry or agency responsible for project implementation (Rogger, 2018). Elected state governors also have a high level of influence over both the budget drafting but also can shift public expenditure allocations away from budgeted allocations. Mogues and Olofinbiyi (2020) find that the state ministry of finance prioritizes allocations for those projects that are a governor’s priority. A similar relationship exists at the local government area (LGA) level whereby the local government chairperson has the final say on expenditure assignments.^8^

India has 28 states as well as eight union territories (UTs). States have their own elected executive (chief minister) and legislature, with autonomy to formulate laws and concurrent representation in the national parliament. The UTs are smaller than states and can be further divided into those that have their own elected legislature, such as Delhi and Puducherry, and those that are centrally controlled by the central government with no representation in Parliament. The Constitution delineates among union, state, and concurrent responsibilities with healthcare being a mandate of the states. However, there are large-scale flagship programs run by the central government, such as the National Rural Health Mission, and the Integrated Child Development Services (ICDS), and insufficient state revenue continues to give the central government a higher degree of influence in implementation at the state level (Sahoo, 2016).

This large number of veto players was viewed positively in India by some in terms of more entry points into the policy sphere: “Government is multi-centered so there is always a possibility of engagement at some level” (I2). When asked about nutrition achievements, several respondents focused specifically on subnational achievements, including food supplementation in Odisha (I12), linking food fortification with the public distribution system in Gujarat (I16), and the adoption of new maternal nutrition guidelines in Bihar (I30). The subnational setting does, however, require advocates that work in different parts of the country to be more adept at multi-level engagement and understanding different priorities of state governments. As one respondent noted, “Earlier, [advocacy] organizations only needed to approach the central government, but now they need to approach the states and states differ in their nutritional deficiencies so what needs to be advocated varies” (I5). Another highlighted the practicalities of *de-jure* and *de-facto* federalism, underscoring that the authority needs to be matched with resources, noting that one challenge for the enabling environment is that it is “excessively centralized and opaque decision making. The way the National Nutrition Mission was conceived but then finally implemented leaves much to be desired. Centre is unable to decentralize and strengthen its federal structure” (I15). In other words, instead of devolving responsibilities effectively to the states in relevant nutrition areas where they legally have a functional role, the national government is perceived as making nutrition decisions unilaterally in some cases.

**Fig. 1:**
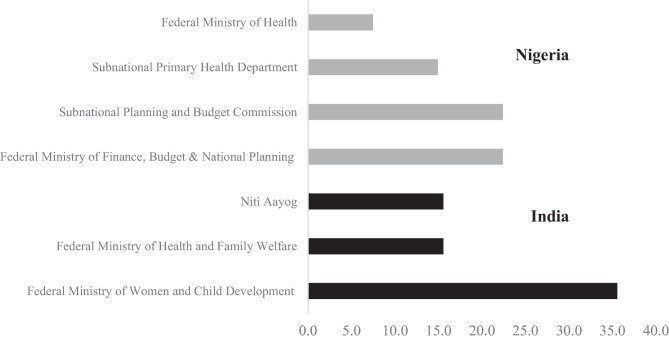
Top most important government institutions for nutrition policy (% of responses)

Beyond the veto players delineated by federal and state functions, there are also a range of government ministries, departments, or agencies involved directly in nutrition policy. Figure 1 highlights the institutions that were collectively identified by a majority of all respondents in each country as the most important. In India, stakeholders identified about a dozen government institutions that they viewed as the primary actors for formulating and implementing nutrition policy. The main actor identified is the Ministry of Women and Child Development (MWCD), followed by the Ministry of Health and Family Welfare (MoHFW) and Niti Aayog, the government’s public policy think tank founded in 2015 to replace the Planning Commission. This largely reflects the budget levels that MWCD and MoHFW allocate to nutrition, which far exceeds any others.^9^ Notably, the range of key actors for nutrition policy and for IYCF policy are relatively similar while those for food fortification substantially differ. In that regard, the Food Safety and Standards Authority of India (FSSAI) is by far seen as the most important actor.

By contrast, in Nigeria, the federal and state ministries for budget and national planning were deemed more important than the Federal Ministry of Health (FMoH). This likely reflects that the National Committee on Food and Nutrition (NCFN), which is the technical arm of the National Council on Nutrition (NCN), is housed within the Federal Ministry of Finance, Budget and National Planning (FMFBNP). On the one hand, the delegation of coordinating responsibilities to a powerful ministry or agency, especially one that oversees spending, is important for implementation. On the other hand, there is some concern that such an important coordinating role has been ceded to a ministry—FMFBNP—that lacks technical knowledge on nutrition (N25). The fact that the Scaling Up Nutrition coordinator is located within FMoH while the NCFN is within the FMFBNP sometimes has been viewed as a deterrent to cooperation and congruence of objectives (Transform Nutrition, 2020).

This ministerial focal point may either be driven or reflective of the fact that many of the nutrition organizations in Nigeria noted that their main emphasis was on budget and finance tracking for nutrition investments. By contrast, in India, budget tracking was not mentioned as a major advocacy or network activity of any advocacy organizations in the sample. Instead, likely reflecting the ministerial focal point there, the emphasis has been on the first 1,000 days, food rights, and the state’s responsibility to enhance welfare outcomes.

Many coordinating bodies exist in both countries on nutrition issues. In terms of institutional coordination, the NCFN and NCN are major modalities at the federal level for nutrition actors in Nigeria. Sub-nationally, the State Committee on Food and Nutrition (SCFN) was established in each state’s planning office while a Local Government Committee on Food and Nutrition (LGCFN) was created in the office of the Vice Chairman of each local government area. In India, the Poshan Mission, which is a multi-ministry initiative led by the MWCD since 2018, was established to coordinate across ministries and ensure better monitoring of goals. Many respondents pointed to the launch of Poshan as one of the key achievements over the last few years that has helped increase awareness of policymakers about child nutrition (I1,2,6, 10, 21, 29, 31). Yet, coordination does not always mitigate competition over scarce resources that ministries require to retain relevance and autonomy over their own individual programming. This was apparent in debates in 2020 when the Ministry of Finance refused to allocate more resources to the MWCD for its Supplementary Nutrition Program, noting that it was already giving MWCD money to run the Poshan Mission and this was otherwise a duplication of resources (Das Gupta & Nair, 2020). There are several other bodies that have been established to facilitate multi-sectoral coordination around nutrition issues in India, including the State Nutrition Missions, and State Rural Livelihood Missions.^10^

We focused on mechanisms at the federal and sub-national level and asked respondents to rank the efficacy of the bodies from 1 (least effective) to 5 (most effective) and, due to expected response bias, present average assessments according to those who are members versus those who are not. There are several conclusions from the findings presented in Table 3 below. First, as seen in the final column, a significant share of respondents in both countries did not know about these coordinating mechanisms and bodies. This is particularly worrying given that the respondents are all highly involved in the nutrition policy sphere. Second, in almost all cases, members of the coordinating bodies were more positive than non-members, suggesting that there is a challenge in convincing outsiders about the value of these entities. The one exception in India is with respect to ICDS, which was ranked the lowest of all entities by its members despite being the body with which most respondents were familiar and a member. This may reflect that it has been in existence for many decades, compared to for instance, the National Health Mission (Poshan), and therefore respondents may be drawing on a longer experience of the body’s performance.^11^ Similar dynamics were observed in Nigeria where the NCFN was viewed more positively among non-members. Again, this may be because the NCFN, which was first established in 1990, is among the oldest bodies listed. While members may be aware of its failings, non-members may recognize it as the main coordinating actor within the FMFBNP. Third, coordinating mechanisms at the subnational level are viewed as modestly more effective by their members. In India, the highest ranked body were the State Nutrition Missions while in Nigeria, the State Committees on Food and Nutrition were viewed the most positively.

**Table 3:** Average ranking of coordinating bodies and mechanisms

**India**	**Member average**	**Total members** **(N)**	**Non-member average**	**Total non-members (N)**	**Don’t know** **(N)**
A. National Health Mission	3.2	11	3.0	14	11
B. State Nutrition Missions	3.4	15	2.4	8	13
C. State Rural Livelihood Mission (UP)	3.0	2	2.7	12	22
D. State Level Steering Committee	3.0	10	2.6	10	16
E. District Nutrition Committees	3.3	10	2.6	11	15
F. Integrated Child Development Services	2.8	18	3.0	9	9

**Nigeria**	**Member average**	**Total members** **(N)**	**Non-member average**	**Total non-members (N)**	**Don’t know** **(N)**
A. National Council on Nutrition	2.5	11	2.5	29	26
B. National Committee on Food and Nutrition	2.6	15	3	27	24
C. State Committee on Food and Nutrition	3.3	32	2.4	17	17
D. National Fortification Alliance	2.4	9	2.2	24	33
E. Universal Salt Iodization Taskforce	3	7	2.5	24	35
F. Micronutrient Deficiency Control Taskforce	3	11	2.3	18	37
G. IYCF Taskforce	2.8	14	2.5	23	29
H. Governors’ Forum	–-	–-	1.8	33	33

Finally, none of the coordinating bodies is largely perceived as highly effective, even by members. This may reflect the broader sense that the existence of inter-ministerial coordinating bodies is not sufficient for mitigating the tensions over budgets and mandates that can still exist across government agencies working in the same domain. Indeed, in India, one respondent noted, “There is no nodal ministry for nutrition, several ministries are involved…It is very difficult to bring all these ministries on to one platform” (I10). Similarly, in Nigeria, one advocate lamented, “From the government side, the main point of disagreement is who really leads nutrition. About the point I made earlier about everyone coming to terms with the multisectoral nature of nutrition, I will say from the government side, they know that but in their implementation or the way they still view nutrition I don't think they really understand how that needs to work, so you still see a lot of the time people say ‘oh nutrition belongs to ministry of health,’ ‘oh agriculture has a more important role to play for nutrition’; so you still see that kind of tussle” (N62).

**Fig. 2:**
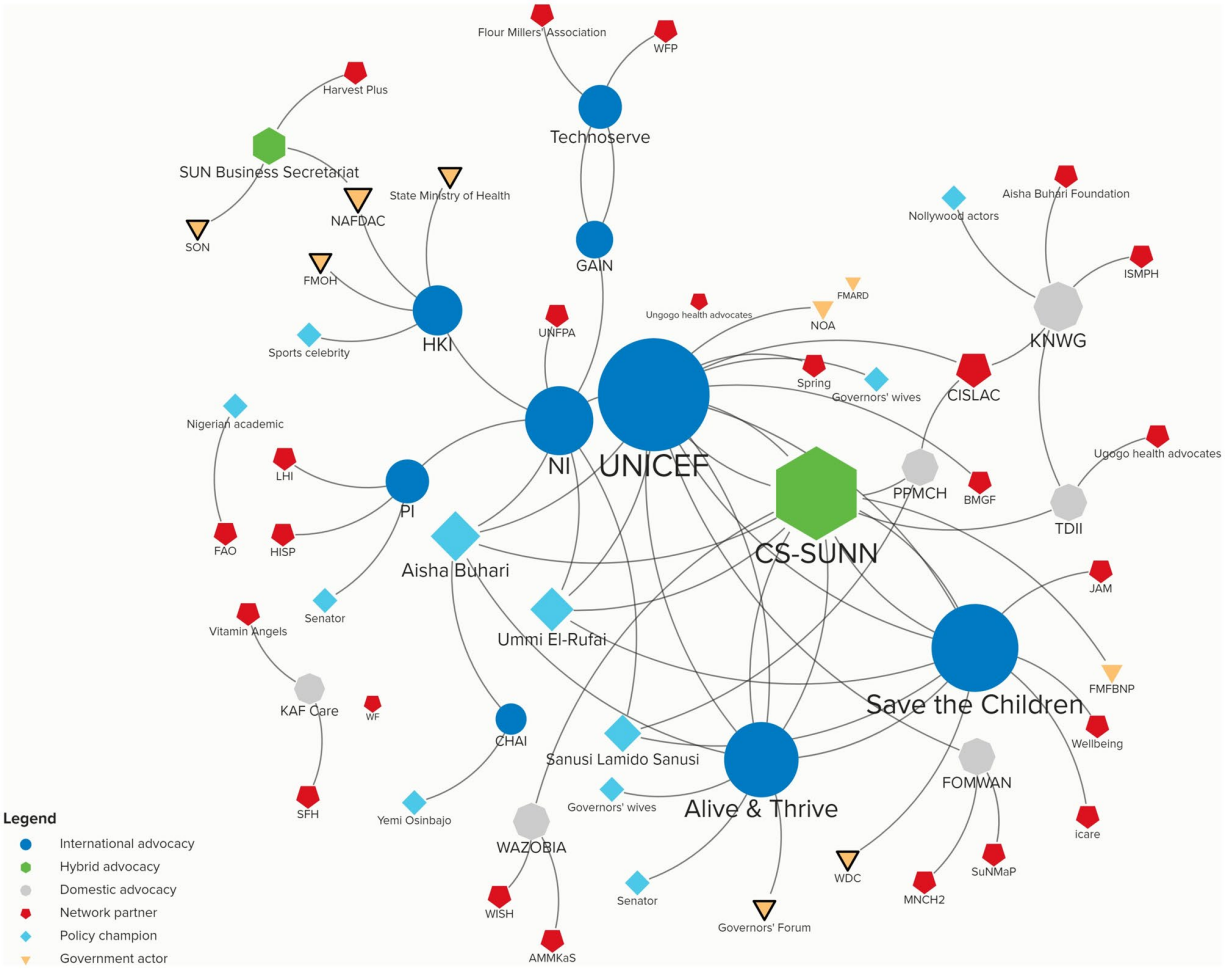
Nutrition advocacy landscape in Nigeria. Notes: Stakeholder analysis conducted using Kumu software

**Fig. 3:**
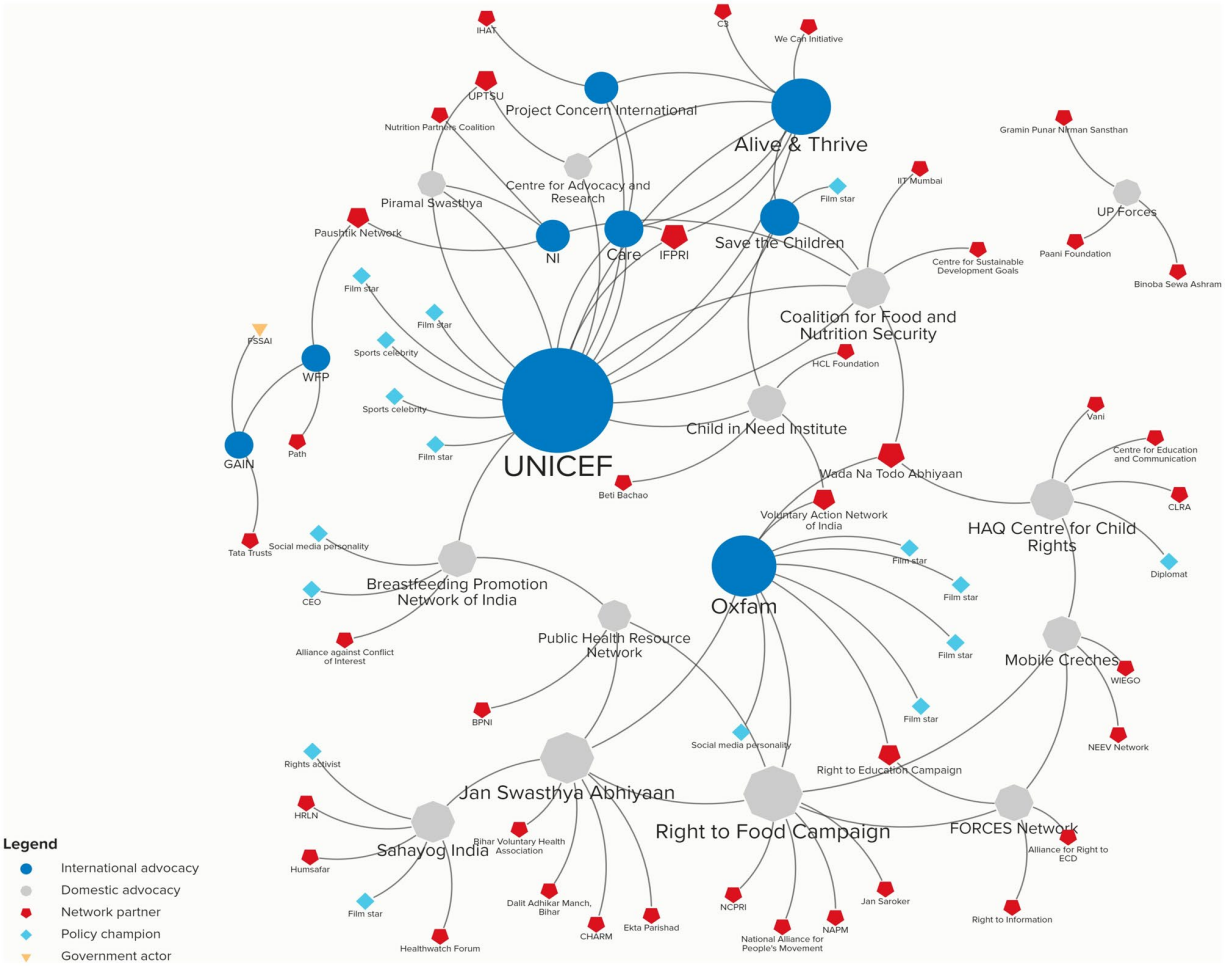
Nutrition advocacy landscape in India. Notes: Stakeholder analysis conducted using Kumu software

In order to assess brokers and policy champions, we asked all of the advocacy organizations in the sample (N = 23 and 25 in Nigeria and India, respectively) to name up to three organizations with which they partner most frequently to advance their objectives. Although this phrasing can lead to an underestimation of total network members, imposing a ceiling reduces the likelihood of respondents listing large numbers of minor partners. Figures 2 and 3 provide an overview of the advocacy landscape in the two countries. The size of the shapes approximates the “degree centrality,” which is a common metric used in social network analysis and involves counting the number of connections attached to each organization (Rice & Yoshioka-Maxwell, 2015). Shapes that are larger are considered more “central” since that indicates more organizations have connections to that organization than to others; this offers a measure of brokerage. The shape and color of the organization indicates whether they are international, domestic, or hybrid advocacy groups, policy champions, government actors, or network partners. The latter category refers to those who were not interviewed but were identified by advocacy organizations as among their top three partners.

Figures 2 and 3 highlight several important features. First, despite having almost an equivalent number of advocacy organizations interviewed in each country, the advocacy networks in India are denser than in Nigeria. In the latter, 48 total actors are identified in the network connected by 18 nodes while in India, there are 63 network partners and 28 nodes. Second, certain advocacy actors are central in both contexts, such as Alive & Thrive, Save the Children, and UNICEF. This likely has also influenced their perceived higher efficacy by Nigerian policymakers (Resnick et al., 2022). Third, India’s domestic nutrition advocacy organizations are more prominent and demonstrate greater network engagement than in Nigeria, where externally headquartered advocacy groups play a more visible role. The prominence of externally-based advocates in Nigeria as brokers may influence the perceived lower degree of national ownership over nutrition policy there (see below). By contrast, in India, several domestically-based organizations demonstrate substantial network engagement with both other local partners and major international ones.

The existence of other nutrition champions is pivotal for advocates to advance their messages and amplify their positions with different audiences. As seen from Figs. 2 and 3, advocates in both countries do indeed draw on such champions. However, in Nigeria, several policy champions were repeatedly identified by advocates as critical partners. Such advocates are predominantly those who hold a great deal of political influence. Among government and donor actors, Hajia Ummi El-Rufai, who is the wife of the Kaduna state governor, was identified as the most important policy champion in Kaduna. She established the Kaduna State Emergency Nutrition Action Plan (KADENAP) in 2017 to fast track activities of Ministries and Departments that deal with nutrition, women, and child health and to ensure synergies rather than duplication of efforts, and she directly attributed UNICEF statistics on levels of malnutrition in Kaduna as the main motivator for her interest in this issue (Kaduna State Government, 2017). At both the federal level and by Kano state respondents, the former Emir of Kano, Dr. Lamido Sanusi, was identified as the most important champion. The wife of the former president, Aisha Buhari, and the spouses of several governors were further viewed as champions on IYCF. In India, policy champions are more diffuse; no advocacy organization identified the same champion twice. Instead of having political leverage, most of the champions that advocates work with are famous film actors as well as several sports celebrities and social media influencers. Importantly, in both countries, international advocacy groups with the greatest brokerage role are also the ones that have more ties with policy champions; this suggests some challenges for equitable advocacy between international and domestic advocacy groups.

### Ownership

While the existence of government policies and frameworks for nutrition are essential for guiding action, translating such documents into concrete outcomes on the ground requires government ownership. This was perceived as more problematic in Nigeria than in India. As one stakeholder noted, “There is no systematic advocacy strategy that is owned by [government] agencies that can have an influence, that have financing and that have capacity to execute these strategies in Nigeria at this point in time both at the federal level and the state level” (N30). Another noted that a real barrier to greater impact on nutrition policy was advocates’ limited “ability to really convince the policy makers to be politically committed. They [advocates] have not been able to really drive down the message for government to really have that political will” (N66).

When respondents were asked what they perceived as the main hindrances to advocacy organizations’ impacts on nutrition policy in Nigeria, the top two concerns were insufficient funds (31%) and lack of political will (29%). In many cases these are quite interrelated since politicians are those that provide requisite funding. In Kano, one government stakeholder explicitly noted the connection: “Lack of release of counterparts fund by state government, poor resource mobilization by nutrition line ministries and agencies, lack of capacity by members of the SCFN, low level of understanding of nutrition issues by the legislature” were all identified as barriers to advocacy efficacy (N23). Recent research by Adeyemi et al. (2023) on Nigeria’s enabling environment for nutrition similarly concluded that the government’s rhetorical commitment to nutrition was not matched with commensurate financial expenditures.

Because funding is such a binding constraint, the donor community is seen as exercising considerable leverage. For instance, one respondent noted, “The issue that we have is the fact that the interventions are donor driven, and the donors have their mandate, and they have their limitations. So, they can come to a state and implement in 5 LGA out of 44 LGAs for example and so scaling-up to the other local governments becomes an issue. We have what you may call pilot activities that are not nationwide. It is just more like a challenge, and it is just that our government is not putting money to scale up interventions” (N34). Although donors are certainly active in India as well on nutrition issues, the bottlenecks to advocates’ impacts on nutrition policy were primarily tied to issues of ideology—particularly reflecting around the issue of eggs discussed earlier—as well as inter-ministerial coordination, and lack of consultation with stakeholders on the ground. Donor interference was not identified as a major challenge there by respondents.

**Fig. 4:**
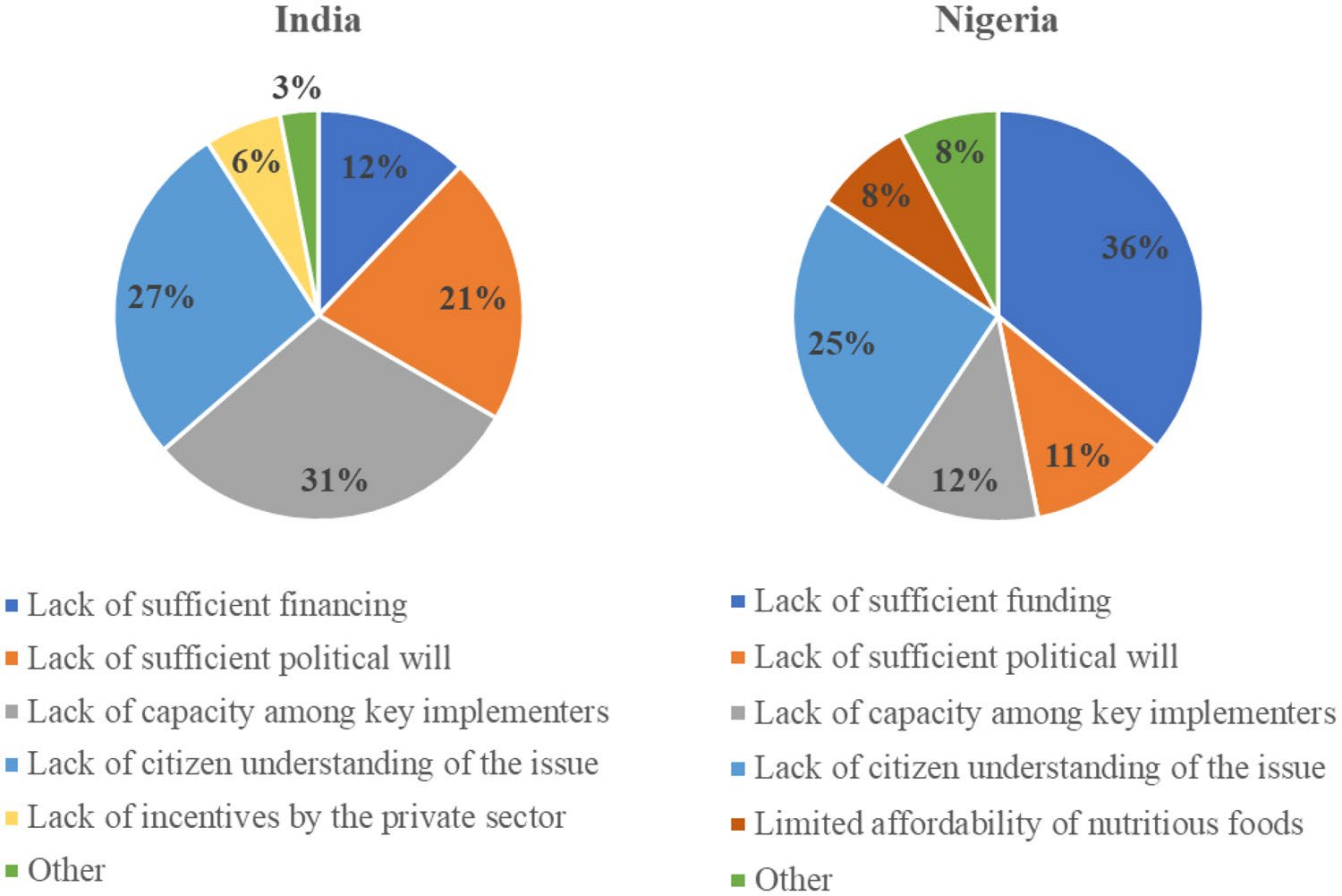
Barriers to greater progress with IYCF

Some of these patterns are reflected through a more fine-grained analysis of IYCF and LSFF. In both countries, respondents were asked to rank, on a scale ranging from 1 to 10, how important they believe that IYCF was to policymakers. The rankings did not vary substantially between the two countries, averaging at 4.59 in Nigeria and 4.35 in India. When there was a specific focus on barriers to IYCF, insufficient funding was again a key concern in Nigeria, with the plurality of respondents noting it was the main barrier to greater progress. This is linked to a concern that donor influence really drives policy efforts in the country. As one respondent noted when asked to elaborate on how important s/he thought IYCF was to the current government, “Not so important. If it is important, they will not wait on donor(s) before they do what they should do” (N02). In India, funding was less problematic than insufficient capacity of implementers and low citizen understanding of the issue (see Fig. 4).

**Fig. 5:**
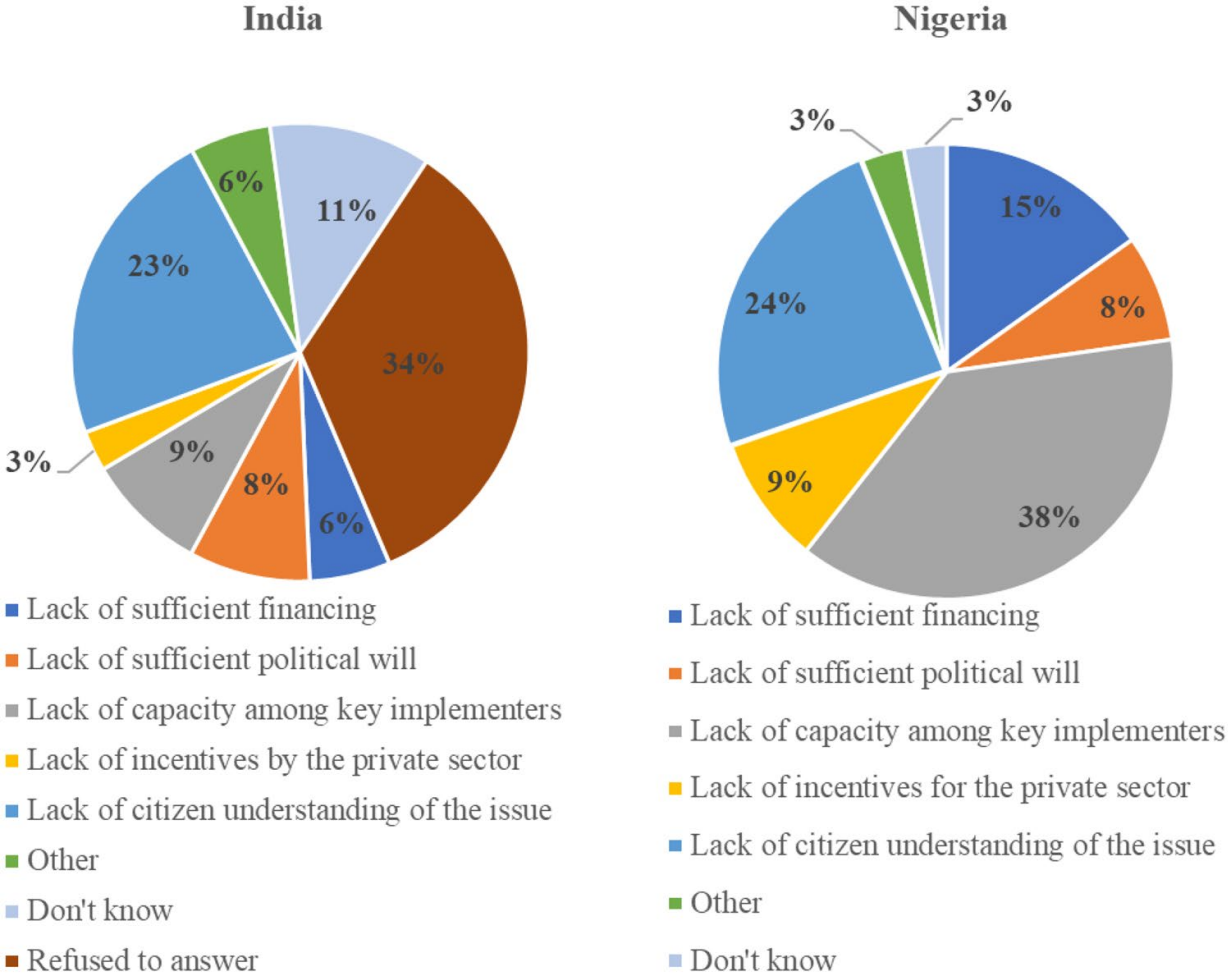
Barriers to greater progress with LSFF

The same scaling exercise was repeated with regards to LSFF, and the findings underscored the importance of considering different dimensions of nutrition policy. In this regard, LSFF was viewed as less important than IYCF in Nigeria, averaging 3.5, compared with a 4.2 in India. Figure 5 highlights that somewhat different barriers are perceived vis-à-vis progress with LSFF. Lack of capacity among key implementers becomes a primary issue in Nigeria. Again, the influence of external actors in this policy domain of LSFF was pronounced: “Nothing seems to be happening in this space, everything is donor-driven. The policy makers are not aware that there is a problem, or the policy makers are not making the right kind of policies or move or getting financing for this” (N30). “If donors do not give them money to do monitoring and enforcement [for LSFF], they don't do it” (N55). “I don't see the impact of policy makers in the area of food fortification in the sense that most of the food fortification activities are mostly private sector and donor driven. I don't see a lot of influence or participation from policy makers” (N62). This somewhat reflects the empirical pattern in Nigeria on LSFF; despite the passage of mandatory standards for fortification for several food vehicles in 2002, several studies uncovered insufficient compliance after 2010 (Aaron et al., 2017; Ogunmoyela et al., 2013). Commitment was revamped through the 2016 Future Fortified Conference during which BMGF committed $10 million for LSFF in the country, parts of which were targeted at funding meetings of the National Fortification Alliance.

Interestingly, many more respondents in India—particularly nutrition advocacy organizations—conveyed that they did not believe in LSFF and refused to identify barriers to greater progress with LSFF. Instead, respondents noted that it was “centrally-driven,” largely by Niti Aayog and FSSAI. The second most selected option for key hindrances to LSFF in India was a lack of citizen understanding.

Another dimension of ownership is the existence of policy mechanisms that help condition and filter advocacy actions. Stakeholders in India pointed frequently to aspects of the social contract between citizens and the state on malnutrition, which underlies many of the country’s large-scale entitlement programs. The concept of the social contract was elevated in 2013 when the Indian Congress Party, convinced by several civil society groups, such as the Right to Food Campaign, passed the NFSA (Lindgren & Lang, 2022; Nisbett et al., 2015). As noted earlier, the NFSA enshrined the right to food by conferring statutory support for several existing programs, such as the mid-day meals scheme that provides free lunches to school children, ICDS, and the PDS. These programs provide an effective lever for advocacy engagement for both IYCF and LSFF, with several respondents noting opportunities for linking nutrition supplements to the ICDS and also the opportunity of enabling ICDS beneficiaries to gain priority within the country’s Mahatma Gandhi National Rural Employment Guarantee Act (MGNREGA), which provides wage employment to rural households (I12, I15, I18).

In Nigeria, there are far fewer social entitlement schemes.^12^ Notably, however, one of the main entitlements related to child nutrition is the inclusion of maternity leave at all levels that is specified in the National Policy on Food and Nutrition (NPFN). For advocates interested in IYCF, and particularly exclusive breastfeeding for six months, the maternity leave policy became a tractable point of entry for policy reform. Organizations such as UNICEF, Alive and Thrive, and Save the Children drew on the 2009 Nigerian Labor Laws and state-level leave policies to lobby for increases from three to six months of leave with full pay to ensure mothers can provide exclusive breastfeeding to their newborns (FMOH et al., 2019). When asked to identify one main policy achievement of the Nigerian government in the area of nutrition policy, the extension of maternal leave was by far the most cited issue, particularly in Kaduna state where KADENAP, Alive & Thrive, CS-SUNN, and Save Children, worked together to convince the governor—a key veto player—to adopt the extension of the maternity leave policy to six months (Adekunle, 2019).

## Summary and discussion

In comparing the two case studies, several key findings emerged. Overall, there is an active nutrition advocacy community in both countries that benefits from a relatively pluralistic political setting. In such settings, it is more likely that leaders’ re-election relies on retaining public support by ensuring their citizens’ well-being, including in the domains of health and nutrition, and that parties need to convey their policy positions and achievements. If an incumbent party is not receptive to advocacy, an opposition party may prove more amenable. Political parties are more programmatic in India than Nigeria which can create a means for advocates to appeal to leaders based on party aspirations but also can limit discussion of certain policy interventions that fall outside the boundaries of accepted ideology. Legal restrictions on the right to association and the right to expression are inherently repressive for civil society, and nutrition advocacy organizations are no different. While both countries have made overtures to further restrict civic space, the FCRA, CAA, and UAPA in India have already impacted the space for maneuver among nutrition advocacy groups.

Veto players are numerous in each case due to not only each country’s federal structure but also to several relevant lead ministries. In each country, there are both concurrent and exclusive functions between central and state governments over health-related interventions, with LGAs in Nigeria and districts in India also having some responsibilities. More veto players provide more points of entry for advocates but also have the potential to prevent quick progress, result in duplication, or lead to contradictions in policy design and implementation. Furthermore, it can hinder advocates from developing a coherent and targeted strategy. The housing of the main inter-ministerial coordinating body, the NCN and NCFN, in the FMFBNP, results in budget issues taking a more prominent role in nutrition decision making in Nigeria while the Ministry of Child Development is predominantly viewed as the lead body in India. Coordinating bodies, such as Poshan or the NCFN, are fundamental first steps in such contexts, but they require sufficient funding and authority to overcome lingering disputes over ministerial mandates and budgets. In addition, based on the stakeholders interviewed for this study, such coordinating mechanisms are not perceived to be highly effective.

A major difference in the two cases is that in Nigeria, advocates and other stakeholders perceive that many nutrition efforts are not necessarily “owned” but rather externally driven, with minimal lack of sustainable political will. International players also appear to have a more prominent role in Nigeria’s nutrition advocacy landscape compared to India where many more domestically-based organizations are active. India also benefits from having several large-scale entitlement programs that advocates can leverage for interventions, such as the ICDS. In domains where such programs exist in Nigeria, such as on labor law around maternity leave, advocates have made progress where they have found willing policy champions and veto players, such as in Kaduna state.

Table 4 delineates several questions to keep in mind when assessing the enabling environment for nutrition advocacy in the domains of voice, access, and ownership. Answering these questions and deriving corresponding indicators will rely on a combination of both qualitative and quantitative approaches. For instance, information on legal restrictions and the degree of democratic competition can be assessed through extant secondary data provided from databases such as the Varieties of Democracy and the Polity projects (see Springman et al., 2022) while public expenditure data can be mobilized to examine budgetary allocations.^13^ In other cases, qualitative tools will be more useful, such as the stakeholder mapping conducted here, Net-Maps that enable advocates to portray their networking from their own perspective (Schiffer & Hauck, 2010) and Circle of Influence graphics that identify veto players in the policy system (Resnick et al., 2018). In still others, elite semi-structured interviews and discourse analysis can uncover strong ideological stances and political will. Importantly, as the third column of Table 4 suggests, not all of the indicators can be interpreted in a unilinear direction since they each bestow advantages and disadvantages. For instance, even if there is not apparent ownership and government buy-in, advocates still play an important role in pushing the policy agenda forward in important ways.

**Table 4:** Identifying enabling environments for nutrition advocacy

**Domain**	**Indicator**	**Implication**
*Voice*	Does the political system allow for democratic competition?	• In democracies, elections provide a form of accountability that leaders address citizen welfare • However, electoral turnover can disrupt advocates’ momentum with previous leaders
	Are there strong ideological stances of political leaders relevant to nutrition policy?	• Programmatic, ideological parties can demonstrate stronger buy-in to advocates’ policy stances than personalistic ones • However, advocates may be ignored if their position conflicts with party ideology
	Are there legal restrictions on freedom of association?	• Restrictions on freedom of association affect the range of tactics and networks that advocates can mobilize
	Are there legal restrictions on freedom of media?	• Restrictions on freedom of association limit the narratives, frames, and modalities that advocates can use to disseminate their positions
*Access*	How many veto players are there in the nutrition arena that advocates are targeting?	• More veto players offer more entrypoints into the policy arena • More veto players also imply slower decisionmaking and potential for duplication
	Are there coordinating bodies that can mediate access?	• Coordinating bodies can mitigate duplication and increase efficiency where there are many veto players • However, they may not be effective if they do not have sufficient financial backing or represent genuine cooperation
	Who are the brokers in the advocacy network?	• More concentrated brokers can lead to power asymmetries and potentially dominate access to decisionmakers • More brokers ensure fewer power disparities but led to potential fragmentation in the advocacy space
	Are there nutrition policy champions that advocates can leverage?	• Champions can amplify advocates’ messages and create credibility with the broader public • Not all champions are equivalent; the reputation and relationship of champions with relevant veto players is therefore critical
*Ownership*	Does the government demonstrate sufficient political will to improve nutrition policy through sustained budget allocations?	• Operational, rather than just rhetorical, commitment is needed for advocates to make a difference over time • However, advocates can still push forward policy discussions even in the absence of budgetary allocations
	Are there extant policies and programs that advocates can leverage to promote their positions?	• Large-scale social protection or health programs can offer a lever for advocates to address nutrition more effectively than recommending an entirely new policy

Overall, the framework elevates several dynamics that are alluded to in other studies about the enabling environment for nutrition but not necessarily examined in-depth. Several analyses emphasize the importance of politics and governance but often equate these factors to the degree of policy coherence, coordination, ownership, political will, and multi-sectoral framings or the relationships between the state and the food industry (Gillespie et al., 2013, 2015; Hodge et al., 2015). While we have integrated several of these important elements into the framework, there is a deeper political and institutional setting that we uncover here specifically through our voice and access domains. In particular, political regime types, veto players, and partisan ideology can equally affect the space for and nature of civic engagement, as can power asymmetries among network brokers.

## Conclusions

Nutrition advocates often have been faulted for not understanding the broader policymaking process outside their area of expertise (Balarajan & Reich, 2016; Cullerton et al., 2016).

However, by drawing on more than 100 interviews with advocates and other stakeholders, this paper demonstrates that there is a strong awareness of what factors have helped or halted progress on advocacy efficacy. In doing so, it highlights that nutrition advocacy is tied to a set of broader factors that are often outside the nutrition space. While nutrition advocacy has been incorporated into extant frameworks as one of many important determinants of nutrition policy outcomes (Shiffman & Smith, 2007) and seen as a component of the enabling environment (Adeyemi et al., 2023), we highlight that governments also shape the regulatory, institutional, political, and financial setting in which advocacy groups—for nutrition and beyond—can operate. Without recognizing this, we only gain a partial understanding of when and why nutrition advocates succeed or fail.

Even in two countries with a variety of institutional and nutrition similarities, we found that the dynamics around voice, access, and ownership vary significantly. Although the analysis is focused on IYCF and LSFF, these three domains are equally relevant to advocacy organizations operating in other policy spheres, including environmental and gender policies. For instance, restricted voice through defamation regulations and closures of CSOs have inhibited environmental advocacy across Asia and Latin America (Le Billon & Lujala, 2020). Similarly, the high barriers to access for amending the constitution in Nigeria have affected the success of gender rights advocates; national senators, state assembly members, traditional authorities, and the president all need to assent in order to adopt a series of gender bills to improve women’s equality (Adetayo, 2022). As such, by drawing on comparative experiences around this framework and deriving broader generalizations, both funders and advocacy organizations can have a better and more realistic understanding of the feasible impact they can achieve in a given policy system—whether for nutrition or otherwise—and target their advocacy strategies accordingly.

## Data Availability

The data generated and analysed during this study arenot publicly available due to the sensitivity of the topic and confidentiality concerns. But, anonymized, selective data will be made available from the corresponding author on reasonable request.

## Appendix 1: Interviewees in Nigeria and India

**Table:**

**Stakeholders, Nigeria**	**Stakeholders, India**
Agricultural Department, Bichi LGA	Alive & Thrive (2)
Agriculture and Forestry Sector, Giwa LGA	BMGF
Agriculture and Natural Resources Department, Wudil, LGA	Breastfeeding Promotion Network of India
Ahmadu Bellow University, Zaria	Care India
Aliko Dangote Foundation	Centre for Advocacy and Research
Alive & Thrive FHI 360	Child in Need Institute
Bayero University	Coalition for Food and Nutrition Security
Civil Society-Scaling Up Nutrition in Nigeria (2)	Confederation of Indian Industry
Clinton Health Access Initiative	Directorate Integrated Child Development Services, Social Welfare Department
Department of Agriculture, Kachia LGA	FORCES Network
European Union Delegation	GAIN India
Express Radio Kano	HAQ Centre for Child Rights
Federal Competition and Consumer Protection Commission	International Institute of Population Sciences
Federal Ministry of Agriculture and Rural Development	Jan Swasthya Abhiyan and Right to Food Campaign
Federal Ministry of Finance, Budget, and National Planning	Mobile Creches
Federal Ministry of Health	MS Swaminathan Research Foundation (MSSRF)
Federation of Muslim Women’s Associations in Nigeria	Niti Aayog (2)
Food and Agricultural Organization	Nutrition International
Global Alliance for Improved Nutrition	Oxfam India
Helen Keller International	Oxford Policy Management
Kaduna Planning and Budget Commission	Piramal Swasthya
Kaduna State Agricultural Development Agency	Project Concern International (2)
Kaduna State Emergency Nutrition Action Plan	Public Health Resource Network
Kaduna State Media Corporation	Right to Food Campaign
Kaduna State Primary Health Care Development Agency	Sahayog India
Kano Ministry of Health	Save the Children
Kano Ministry of Planning and Budget	The Soyabean Processors Association of India
Kano Nutrition Working Group	UNICEF (2)
Kola and Funke Care Foundation	UP FORCES – Secretariat Vigyan Foundation
Ministry of Agriculture	USAID
National Orientation Agency	World Bank
Nutrition consultants (3)	World Food Program
Nutrition International	
Partnership for the Promotion of Maternal and Child Health in Kano State	
Plan International	
Primary Health Care Department, Giwa LGA	
Primary Health Care Department, Kachia, LGA	
Primary Healthcare Department, Bichi LGA	
Primary Healthcare Department, Wudil LGA	
Save the Children (2)	
Standards Organization of Nigeria	
State Primary Health Care Management Board	
SUN Business Network	
Technoserve	
Transparency and Development Information Initiative	
UK Department for International Development	
UNICEF (2)	
Wazobia International Women and Children Foundation	
World Bank	

For organizations where more than one respondent was interviewed, the number in parentheses indicates the number that was interviewed

## Footnotes

1 See http://sdg2advocacyhub.org/suportsdg2

2 A separate paper focuses specifically on advocacy organizations.

3 See, however, Teets (2018) for an insightful analysis of the different tactics advocates may use in authoritarian systems.

4 The full questionnaire is available upon request to the authors.

5 Eighteen government officials from relevant ministries and agencies that oversee nutrition in India were contacted but only three agreed to be interviewed.

6 Besides length of experience with their organizations, advocates’ age, ethnicity, caste, and gender are other important characteristics that future analyses should consider since they can potentially affect individuals’ advocacy strategies. We thank an anonymous reviewer for highlighting this important point.

7 A summary of provisions from the 2017 bill can be found here: https://spacesforchange.org/summary-of-the-ngo-bill/

8 The local government chairperson may be elected or appointed as a caretaker committee. This varies substantially depending on the state (Fetzer & Kyburz, 2024).

9 See Poshan Abhiyaan website: http://poshanabhiyaan.gov.in/#/. (Accessed December 1, 2021).

10 State Nutrition Missions serve as an overarching governance body to improve coordination, communication, and strategic planning for nutrition. Maharashtra was the first state to set up a State Nutrition Mission in 2005 which served as a model for other states. (https://r4d.org/projects/improving-nutrition-women-children-india-state-nutrition-missions/).

11 Respondents could also identify other bodies they felt were relevant for either multi-sectoral or multi-level coordination. These included State Food Commissions, Water and Sanitation Committees, Bihar Nutrition Alliance (Bihar), and the State Plan of Action for Children Coordinating Committee (Uttar Pradesh).

12 Nigeria did launch its Home Grown School Feeding Program in 2016 to offer lunches to school-aged children but the scale of the program is not comparable to India’s ICDS (Agu et al., 2023).

13 More information on the V-Dem and Polity data initiatives can be found at https://www.v-dem.net/ and https://www.systemicpeace.org/inscrdata.html, respectively.
